# A novel HPV 16 L1-based chimeric virus-like particle containing E6 and E7 seroreactive epitopes permits highly specific detection of antibodies in patients with CIN 1 and HPV-16 infection

**DOI:** 10.1186/1743-422X-8-59

**Published:** 2011-02-09

**Authors:** Alberto Monroy-García, Miguel A Gómez-Lim, Benny Weiss-Steider, Georgina Paz-de la Rosa, Jorge Hernández-Montes, Karyna Pérez-Saldaña, Yessica S Tapia-Guerrero, Mariel E Toledo-Guzmán, Edelmiro Santiago-Osorio, Héctor I Sanchez-Peña, María de Lourdes Mora-García

**Affiliations:** 1 Unidad de Investigación Médica en Enfermedades Oncológicas. IMSS, CMN SXXI, México; 2 Centro de Investigación y de Estudios Avanzados (CINVESTAV), Unidad Irapuato, Guanajuato, México; 3 Laboratorio de Inmunobiología, Unidad de Investigación en Diferenciación Celular y Cáncer. FES-Zaragoza, UNAM, México. Laboratorio 3, PB, UMIEZ. Campus II. Facultad de Estudios Superiores Zaragoza, UNAM, Batalla 5 de mayo s/n, Col. E. Oriente, Esquina Fuerte Loreto, Iztapalapa, CP 09230, México, DF., México; 4 Laboratorio de Hematopoyesis, Unidad de Investigación en Diferenciación Celular y Cáncer. FES-Zaragoza, UNAM, México; 5 Departamento de Ginecología. H.G.Z No. 2-A Troncoso, IMSS

## Abstract

**Background:**

The presence of IgG antibodies to HPV-16 L1-virus like particles (VLPs) in serum has been reported as a result of persistent exposure to the virus and as a marker of disease progression. However, detection of VLP-specific antibodies in sera does not always indicate a malignant lesion as positive results may also be due to a nonmalignant viral infection. Furthermore, malignant lesions are associated with an increased antibody titer for E6 and E7 proteins. The aim of this study was to develop an ELISA using a novel chimeric virus-like particle (cVLP) encoding an L1 protein fused with a string of HPV-16 E6 and E7 seroreactive epitopes to its C-terminus to be used for detection of HPV-16 specific antibodies in patients with cervical intraepithelial lesion grade 1 (CIN 1).

**Results:**

The sera of 30 patients with CIN 1 who also tested positive for HPV-16 DNA and of 30 age-matched normal donors negative for HPV infection were tested for the presence of IgG antibodies specific for either VLP-L1 (HPV-16 L1), gVLP (derived from Gardasil), or cVLP by ELISA. The cVLP-reactive sera yielded two distinct groups of results: (H) reactivity levels that presented very strong cVLP-specific titers, and (L) reactivity levels with significantly lower titers similar to those obtained with VLP-L1 and gVLP antigens. Additionally, the sera that presented the higher cVLP titers closely matched those that had significantly stronger reactivity to E6 and E7 epitopes. Interestingly, the samples with the highest titers corresponded to patients with the higher numbers of sexual partners and pregnancies. On the other hand only 4 out of the 12 sera that harbored antibodies with VLP neutralizing ability corresponded to the group with high cVLP antibody titers.

**Conclusion:**

We report for the first time that chimeric particles containing HPV-16 L1 protein fused with E6 and E7 seroreactive epitopes enable much better detection of IgG antibodies in the sera of CIN 1 patients positive for HPV-16 infection than those obtained with VLPs containing only the HPV-16 L1 protein. We also found that the sera with higher cVLP antibody titers corresponded to patients with more sexual partners and pregnancies, and not always with to those with a high neutralizing activity. This novel assay could help in the development of a tool to evaluate cervical cancer risk.

## Background

Infection of the genital epithelium with human papillomavirus (HPV) is a common sexually transmitted disease, as well as a significant public health burden in developing countries. Most cervicovaginal HPV infections are clinically inapparent and produce no cytological abnormalities. However, persistent infections with certain high-risk HPV strains can cause cervical cancer, which is the second most common cancer in women worldwide and accounts for 250,000 deaths annually [1]. High-risk HPV strains, such as HPV-16, HPV-18, and others, are detectable with sensitive polymerase chain reaction (PCR) methods and are present in more than 95% of all abnormal cervical cytology samples [2]. Notably, HPV-16 accounts for 50% to 60% of all HPV DNA-positive cervical cancers [3]. HPV DNA encodes a range of early (E) functional and late (L) structural capsid proteins that are immunogenic. L1 is the major capsid protein of HPV. The oncogenic E6 and E7 proteins are expressed in tumor tissues at all stages of cancer progression and interfere with p53 and the retinoblastoma gene product to maintain the proliferative status of HPV-infected tumor cells [4].

Studies of the immune response to HPV have progressed slowly, due in part to the lack of suitable reagents for immunologic assays. During the last two decades, serologic studies have been important in understanding the natural history of HPV infections and efforts are ongoing to develop reliable genotype-specific assays. In this context, the observation that HPV capsid proteins generated in eukaryotic expression systems self-assemble into virus-like particles (VLPs) has enabled the use of these particles as reagents for studies of the immune response to HPV [5-9].

A number of studies have demonstrated that human sera can react with HPV VLPs and that this reactivity is largely HPV- and strain-specific [10-12]. In addition, a strong association between HPV-VLP antibody seropositivity and the development of cervical lesions or the progression of these lesions to cervical cancer has been demonstrated [13-15]. Previous epidemiological studies have shown that the presence of HPV-16 VLP-specific antibodies is associated with a 12.5 times increased risk for subsequent development of either carcinoma *in situ *or invasive cervical cancer [16,17]. However, despite data demonstrating these associations, the presence of VLP-specific antibodies is not necessarily indicative of viral infection as antibody detection is strongly asynchronous with infection and the antibody response to HPV proteins does not invariably occur during a natural HPV infection. In fact, it has been reported that humoral immune response to VLPs is induced in about half of women with normal cytology who have HPV DNA present in their cervical epithelium [17].

The presence of antibodies specific for the viral tumor antigens E6 and E7 is considered the best marker for cervical cancer progression. This is because the magnitude of the antibody response is influenced by the stage of disease and the size of tumor mass [18-20]. Optimal detection of antibodies specific for L1 in conjunction with those specific for E6 and E7 antigens may be important in understanding HPV seroconversion in patients with primary lesions, as well as to evaluate the clinical outcome of viral infections and the progression from infection to cervical cancer.

In the present study, we developed an ELISA to detect cVLP-specific IgG antibodies in serum samples from patients with incipient lesions such as cervical intraepithelial lesion grade 1 (CIN 1) that were also positive for HPV-16 infection. This ELISA utilizes a novel chimeric VLP (cVLP) composed of HPV-16 L1 and a string of E6 and E7 epitopes fused to the C-terminus [21]. Interestingly, the cVLPs used in this study enabled significantly improved detection of IgG antibodies in these patients versus the results obtained using VLPs containing only the HPV-16 L1 protein.

## Results

### Detection of IgG antibodies specific for virus-like particles in serum samples

A total of 230 female patients with CIN 1 were analyzed for the presence of HPV DNA by PCR, and only the sera from the 30 patients who tested positive for HPV-16 infection were included in this study. On the other hand, serum samples from 30 women without clinical or molecular evidence of HPV infection were used as negative control for these experiments. The mean age of the experimental subjects was of 32.1(16-43) years while in the normal donors was of 36.4(19-47). The reported mean of sexual partners and pregnancies of the patients was of 1.7(1-5) and 2.5(1-5) respectively, while in the normal donors was of 1.2(1-3) and 1.1(1-3). The sera and clinical data from all participants were taken after their informed consent and according to the ethical and confidentiality requirements concerning work with human samples of the institutions involved.

**Figure 1:**
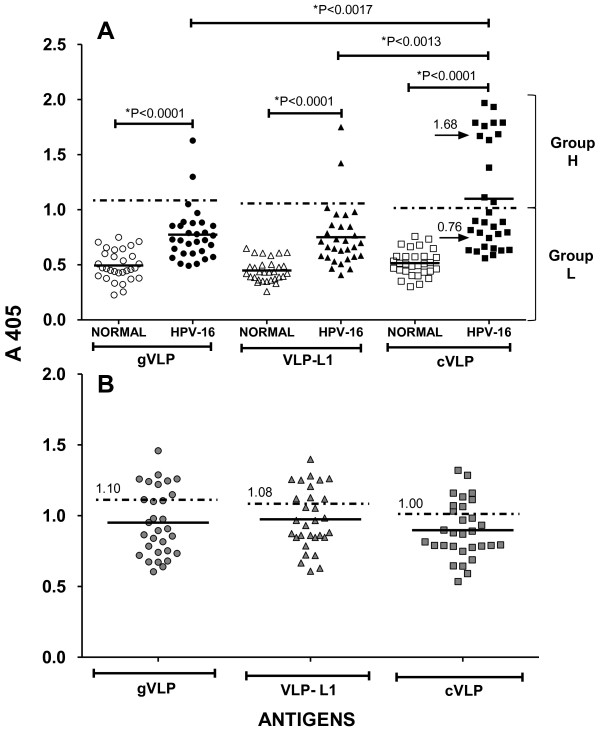
**Seroreactive response of patients with cervical intraepithelial neoplasia grade 1 (CIN 1) and HPV-16 infection to various virus-like particles (VLPs)**. **(A) **IgG antibody responses to VLPs derived from: the commercial vaccine Gardasil^® ^(gVLP, circles) containing HPV-16, -18, -6 and -11 serotypes; plant-based VLPs composed by HPV-16 L1 protein (VLP-L1, triangles); or chimeric particles containing the HPV-16 L1 sequence and sequences encoding E6 and E7 known antigenic peptides fused to the C-terminus (cVLP, squares), were determined in sera from women with no evidence of HPV infection (normal, open symbols), and in sera from patients with CIN 1 and HPV-16 infection (HPV-16, filled symbols). Results are divided into two specific groups of titers: a high-titer group (H) and a low-titer group (L). The mean absorbance distribution of each group is represented by a solid line. Values of the mean absorbance distribution of the H and L groups are indicated by arrows. **(B) **Shown are the mean absorbance distribution of the control positive group and the cut-off values for high seroreactivity at A 405 (indicated by horizontal dashed lines and numbers) in response to gVLP (gray circles), VLP-L1 (gray triangles), and cVLP (gray squares). *Indicates difference between groups is statistically significant. P-values were calculated using Wilcoxon signed-rank test and Student's t-test.

**Figure 2:**
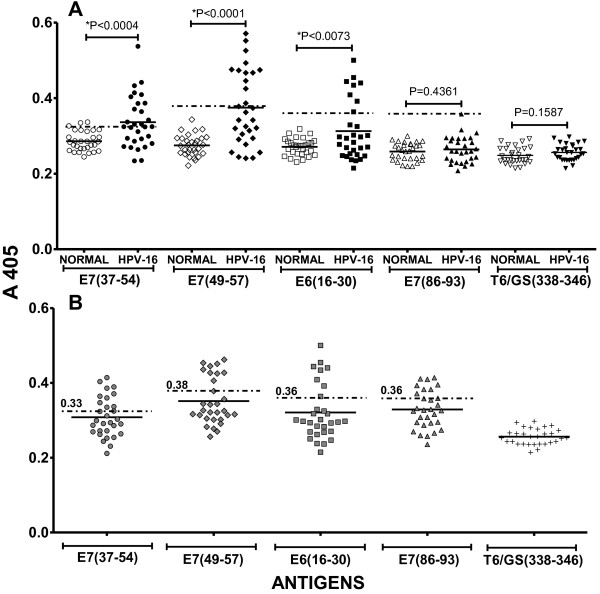
**Seroreactive response of patients with CIN 1 and HPV-16 infection to HPV16 E6 and E7 antigenic peptides contained in cVLPs**. **(A) **The IgG antibody response in sera from donors without evidence of HPV infection (normal) and in sera from patients with CIN 1 and HPV-16 infection (HPV-16), against antigenic peptides derived from HPV-16 proteins was determined by ELISA. The HPV-16 peptides were: EIDGPAGQAEPDRAHYNI (E7 37-54), RAHYNIVTF (E7 49-57), PRKLPQLCTELQTTI (E6 16-30), and TLGIVCPI (E7 86-93). Additionally, the peptide MQYEIINYM T6/GS (338-346), derived from the polymerase accessory protein, was used as a negative control. The mean absorbance value of each group is represented by a solid line. **(B) **Shown are the mean absorbance distribution of the control positive group and the cut-off values for high seroreactivity at A 405 (indicated by horizontal dashed lines and numbers) in response to the seroreactive peptides E7 37-54 (gray circles), E7 49-57 (gray rhombus), E6 16-30 (gray squares), E7 86-93 (gray triangles). The seroreactivity to the irrelevant peptide T6/GS(338-346) (tails) is also depicted. *Indicates difference between groups is statistically significant. P-values were calculated using Wilcoxon signed-rank test and Student's t-test.

The presence of IgG antibodies specific for three types of VLPs was determined by ELISA. Two of the VLPs were plant-derived and composed of either the HPV-16 L1 protein (VLP-L1) or a chimeric particle containing HPV-16 L1 and a string of E6 and E7 known antigenic peptides fused to the C-terminus (cVLP). The third type of VLP was derived from the commercial vaccine Gardasil^®^, and contained the serotypes HPV-16, -18, -6 and -11. As expected, the mean absorbance value for the levels of IgG antibodies detected in sera from the CIN 1 patients using either gVLP, VLP-L1, or cVLP were higher than those from normal donors (Figure 1A). In the same experimental assay we included a control group selected from a previous study [22], consistent of 30 samples with reactivity of IgG antibodies for VLPs. Taken into consideration the mean absorbance distribution of the sera reativity +3 SD of this control group to gVLP, VLP-L1 and cVLP, the cut-off values for high reactivity were of 1.1, 1.08 and 1.0 respectively (Figure 1B). According with these cut-off values, we obtained two distinct absorbance groups with cVLP that we called high (group H) and low (group L) responders with mean absorbance values of 1.68 ± 0.24 and 0.76 ± 0.14 respectively (Figure 1A). It is interesting to mention that the average absorbance of the low responders in group L was higher that that of any of those obtained from the negative control sera. In the high responder group using cVLP we had the serum samples of 11 patients (37%) while 19 patients in the low responder group (63%). When we used VLP-L1 or gVLP we only had 2 serum samples in the higher responder group (7%). Our results thus suggest a more sensitive ELISA assay when using cVLP as compared with those using VLP-L1 or gVLP. We hypothesize that this significant difference is due to presence of E6 and E7 antigenic peptides fused to the L1 protein in the chimeric cVLP particle. Of note, the patients that reported having more sexual partners and pregnancies also presented higher reactivity to the antigenic peptides E6 and E7 as well as to cVLP (Figure 2, Table 1).

**Table 1:** Clinical data and seroreactive response to VLPs or HPV-16 E6 and E7 antigenic peptides in sera from CIN 1 patients.

gVLP (1.10)	VLP-L1 (1.08)	cVLP (1.00)	E7(37-54) (0.33)	E7(49-57) (0.38)	E6(16-30) (0.36)	Sera Neutralizing dilution to VLPs	Number of sexual partners [3.0]	Number of Pregnancies [3.5]
		1.933	0.364	0.475	0.364	-	5	4
		1.790	0.433	0.473	0.392	-	2	3
		1.760	0.337	0.493	0.434	-	2	5
		1.670	0.264	0.466	0.214	-	1	2
1.628	1.751	1.633	0.293	0.342	0.301	1:100	2	5
		1.791	0.311	0.241	0.253	1:100	2	4
1.299	1.423	1.969	0.386	0.425	0.444	-	3	3
		1.686	0.371	0.446	0.500	-	5	4
		1.790	0.299	0.277	0.297	1:100	4	2
		1.113	0.324	0.386	0.269	1:200	2	3
		1.383	0.537	0.571	0.409	-	3	4

Shown are the IgG serum antibody absorbances to VLPs or HPV-16 E6 and E7 antigenic peptides from CIN 1 subjects from Group H (Figure 1). The cut-off values for a high seropositive response to each antigen are shown in parenthesis. Serum dilutions with neutralizing activity to VLPs are also indicated. Absence of neutralizing activity is indicated by "(-)". Mean numbers of sexual partners and pregnancies are shown in square brackets.

### Detection of antibodies with specific neutralizing ability for virus-like particles

Several studies have shown that infection with HPV induces VLP-specific antibodies with neutralizing ability that are maintained over long periods of time [10,23-25]. To verify the specificity and neutralizing ability of these antibodies, hemagglutination inhibition assays (HIA), based on the ability of the antibodies to inhibit the interaction between virus-like particles with cell receptors present in the membranes of erythrocytes resulting in agglutination, have currently been used [21,26,27]. In order to investigate whether antibodies in the serum samples of patients with CIN 1 and infected with HPV-16 have neutralizing activity, we employed an HIA. Serial dilutions of serum samples from either patients with CIN 1 and HPV infection or normal controls were incubated with purified VLP-L1, cVLP, or gVLP, and subsequently with mouse erythrocytes. We found that between 11 and 12 serum samples out of 30 patients (approximately 40%) completely inhibited gVLPs, VLP-L1 s, and cVLPs hemagglutination at 1:100 to 1:400 sera dilutions, while only between 4 to 6 serum samples (approximately 20%) from normal donors inhibited hemagglutination but only at 1:100 dilutions (Table 2). We consider important to mention that less than 50% of serum samples from patients with CIN 1 that had high seropositivity to cVLPs had neutralizing ability for any of the three types of VLPs used in this study (Table 2).

**Table 2:** Frequency of patients with CIN 1 and HPV-16 infection and normal donors who had serum titers of antibodies with neutralizing activity to VLPs.

	SERA	
ANTIGENS	CIN 1 PATIENTS HPV-16+	NORMAL DONORS
	6/30 (1:100)	
**gVLP**	3/30 (1:200)	6/30 (1:100)
	3/30 (1:400)	
	Total 12/30	

	5/30 (1:100)	
**VLP-L1**	4/30 (1:200)	4/30 (1:100)
	2/30 (1:400)	
	Total 11/30	

	5/30 (1:100)	
**cVLP**	4/30 (1:200)	4/30 (1:100)
	2/30 (1:400)	
	Total 11/30	

Shown are results of hemagglutination inhibition assay (HIA) was performed to measure titers of antibodies with neutralizing activity for specific VLP particles in sera from women without evidence of HPV infection (normal) and in sera from patients with CIN 1 and HPV-16 infection. Serum from each person was assayed at various dilutions as described in the Materials and Methods. Frequencies of patients and titers of antibodies with neutralizing activity (in parentheses) for gVLPs, VLPs, and cVLPs are shown.

## Discussion

Persistent HPV-16 VLP serum antibodies are associated with an increased risk of developing either carcinoma *in situ *or invasive cervical cancer. Moreover, the presence of anti-E6 and anti-E7 antibodies is associated with malignant progression of these cancers [28,29]. Optimal detection of antibodies specific for E6 and E7 antigens in conjunction with those specific for VLPs in the incipient stages of the disease may be important to evaluate the clinical outcome of viral infection and the possible progression of this infection to cervical cancer. In order to contribute to the early detection of these antibodies, we utilized a novel plant-derived chimeric virus-like particle (cVLP) composed of HPV-16 L1 and a string of E6 and E7 antigenic peptides fused to the C-terminus [21]. We designed an ELISA using cVLP for the detection of specific antibodies in the sera of subjects diagnosed with cervical intraepithelial lesion grade 1 (CIN 1) and HPV-16 DNA-positive genital tract tissue. With respect to sera from CIN 1 patients, our results demonstrated more sensitive detection of specific IgG antibodies using cVLP versus those obtained with either VLPs containing only the HPV-16 L1 protein (VLP-L1) or with VLPs derived from the commercial vaccine Gardasil (MSD, USA; gVLPs) (Figure 1). In addition, we have obtained that sera of the patients used in this study showed a similar reactivity to Cervarix (Glaxo SK, UK) (0.72 of mean absorbance distribution) when this vaccine was used as heterologous antigen. Interestingly, our results using cVLP could be divided into two distinct groups: one group of serum samples had low titers of antibodies (Group L, Figure 1), levels which were similar to most of the results obtained from ELISAs using VLP and gVLP and indicative of a high specificity to the L1 protein; a second group which had serum samples with very high antibody titers (Group H, Figure 1). The fact that these high antibody titers were detected by ELISAs using epitopes from E6 and E7 proteins in addition to L1 indicate that the specificity was for the E6 and E7 antigenic peptides contained in cVLP (Group H, Figures 1, 2 and Table 1).

The integration of HPV DNA increases oncogene expression at various time points of viral pathogenesis and malignant transformation [30]. Additionally, the presence of antibodies specific for HPV-16 E6 and E7 proteins have often been associated with an increased risk of subsequent carcinoma development [28,29]. Thus, the presence of specific antibodies to cVLPs found in serum samples of the subjects with CIN 1, particularly in Group H, may indicate not only the persistence of HPV-16 infection, but also the possible integration of the viral genome in cervical epithelial cells. Interestingly, cervical brush samples of patients with CIN 1 were not only positive for the presence of E6 and E7 HPV-16 genes as determined by PCR analysis, but were also associated with patients who had a larger number of sexual partners (mean 3) and pregnancies (mean 3.5) (Table 1), compared to control donors samples. In fact, large numbers of sex partners and pregnancies have been reported as major risk factors associated with persistent HPV infection and increased risk of developing cervical dysplasia and cancer [31,32]. Because antibodies specific for HPV-16 E6 and E7 proteins are associated with malignant progression of the disease, and in fact are markers for HPV-associated invasive cervical carcinoma, we hypothesize that the presence of high-titer antibodies specific for HPV-16 E6 and E7 epitopes in Group H patients with CIN 1 (Table 1) may indicate a higher risk of cancer progression [33]. These conclusions have been further supported by experiments involving synthetic peptides and proteins translated *in vitro *or produced in bacteria or baculovirus/insect cell systems. These studies found that the prevalence of anti-E6 and anti-E7 antibodies varies from 16% to 60% in people with cervical uterine cancer, versus only 4% to 20% in normal donors [34-37]. In the present study, the results of ELISAs that contained the peptides E7 37-54, E7 49-57, and E6 16-30 as individual antigens indicated that sera from 8 out of the 11 CIN 1 patients had high titers of antibodies specific for cVLP (Table 1). These results indicate that these three epitopes were the ones bound by the serum IgG antibodies from patients with CIN 1 concomitant to a persistent HPV-16 infection.

Results from several studies have indicated that HPV infection induces highly strain-specific neutralizing antibodies based on the fact that recipients injected with HPV-16, -18, -31, -52 and -58 VLPs develop neutralizing antibodies specific for homologous HPVs efficiently and with very limited cross-neutralization [11]. Furthermore, patients with past and the persistent HPV can be identified by detection of anti-HPV strain-specific neutralizing antibodies. In this study, we found that almost 40% of the patients with CIN 1 and positive for DNA HPV-16 had serum antibodies specific for VLPs and with neutralizing ability, which we determined with a hemagglutination inhibition assay. However, only a very few number of serum samples from patients with CIN 1 (4 out of 30) and high titers of antibodies specific for cVLPs, HPV-16 E6 and E7 peptides had VLP neutralizing activity. We hypothesize that these low levels of antibodies with neutralizing ability in sera from these patients may indicate a lack of sufficient protection against repeated infection with either homologous or heterologous HPV types [38]. Consequently, a persistent HPV-16 infection associated with low levels of antibodies with neutralizing ability may raise the risk of HPV E6/E7 oncogene-cell DNA integration, leading to the initial step in cell immortalization by HPV.

It would be interesting to repeat the present experiments using a large number of serum samples from patients infected with HPV strains other than HPV-16 and with sera from patients with CIN 1 and cervical cancer. We hypothesize that the use of ELISAs using the cVLPs described herein may be useful in monitoring the pre-existing serostatus of women who have been vaccinated with the recently introduced prophylactic VLP-based vaccines. Furthermore, these ELISAs may be useful in evaluating the vaccine response in women with or without preexisting IgG antibodies specific for viral particles, especially those antibodies specific for high-risk viruses.

Lastly, we think that the ELISA described herein may prove to be a useful tool in evaluating the risk of cancer progression in women that have high titers of sera antibodies specific for L1 plus E6 and E7 epitopes. Supporting this idea is the fact that longitudinal studies have demonstrated that women with incident infection who have seroconverted were 5 times more likely to have SIL than women who had not seroconverted [24,25]. Additionally, the persistence of HPV-16 VLP serum antibodies is also associated with increased risk of both carcinoma *in situ *or invasive cervical cancer [16,17], making this novel ELISA a potentially important clinical tool.

## Materials and methods

### Study population and human samples

The study population was selected from patients attending the National Center for Clinics of Dysplasias (CENACLID), General Hospital of Mexico, and the Department of Gynecology H.G.Z No. 2-A Troncoso, IMSS, Mexico City, Mexico. Serum samples were collected between February 2008 and July 2010 and informed consent was obtained from all participants. Human material was handled according to institutional experimentation and safety guidelines. CENACLID provides gynecological services to women referred for colposcopy because of abnormal cytology and women who do not have a history of cervical abnormalities but ask for a routine examination. In the present study, women in both groups were analyzed. All women underwent cytological and histopathological analysis of colposcopy-directed biopsies. Two experienced cytotechnologists independently examined all Pap smears. Samples with an inconsistent diagnosis were excluded from the study. Cytology diagnoses were classified according to the Bethesda System while the histopatological one, of the Cervical Intraepithelial Neoplasia Grade I (CIN I), was according to Richardt [39]. Samples with Cervical Intraepithelial Neoplasia Grade II or III were excluded from the study. All samples underwent molecular analysis by PCR and hybrid capture. Women who were consistently negative for both clinical and molecular tests were considered negative controls. Only patients with CIN 1 who had consistent positive results by PCR were included in the study.

Blood samples, as well as cervical punch biopsy material or cervical cells adequate for HPV DNA measurement, were obtained both from patients with CIN 1 who were also positive for HPV-16 infection and from negative controls. Cervical cells were collected by gently swabbing the ectocervix with a cytobrush (Digene). Cervical cells and biopsied tissues were placed immediately in tubes containing sterile, contaminant-free PBS (Roche) and processed the same day. Serum was isolated from blood samples by centrifugation at 9000 *g *for 15 min. Serum samples were stored at -70°C until tested. Investigators who performed molecular and immunological assays were not aware of the clinical status of samples. To avoid contamination, cases and controls were tested in separate batches.

### Detection of high- and low-risk HPV strains by hybrid capture

The HPV Test Hybrid Capture II kit (Digene) was used to detect high-risk HPV strains (16, 18, 31, 33, 35, 39, 45, 51, 52, 56, 58, 59 and 68) or low-risk strains (6, 11, 42, 43 and 44). DNA (250-500 ng in 20 μl) was extracted from cervical cells or tissues and placed in a tube containing 30 μl specimen transport medium. NaOH-based denaturation reagent (25 μl) was added to each sample and the tubes were mixed vigorously and incubated at 65°C for 45 min. Hybridization and hybrid detection were performed according to the manufacturer's instructions. Carrier DNA and plasmid DNA containing the HPV-16 or HPV-11 genome were used as negative and positive calibrators, respectively, and were run in triplicate with each test. An assay was considered valid only when the results from the negative and positive calibrators showed a coefficient of variation ≤15% and the positive-to-negative calibrator mean values ratio was ≥2.0. The cut-off value for positivity was calculated for each assay and was defined as the mean relative light units (RLU) value of the positive calibrator. Samples that were positive for low-risk types were excluded from this study.

### HPV DNA detection by PCR

All reagents used in the isolation and amplification of DNA were purchased from Gibco-BRL. Biopsies were treated with proteinase K as described elsewhere [40]. DNA was extracted with phenol/chloroform and precipitated with ethanol. HPV DNA was amplified using the general primers MY09 (5'-CGTCCMARRGGAWACTGATC-3') and MY11 (5'-GCMCAGGGWCATAAYAATGG-3') [41], which amplified a conserved 450-bp fragment from the L1 gene. Genomic DNA (100 ng) was denatured by heating to 95°C for 30 s. Annealing of primers was performed at 45°C for 30 s and extension at 72°C for 60 s. The cycle was repeated 30 times. Specific amplification of HPV-16 was achieved by using the primers Pr3 (5'-GTCAAAAGCCACTGTGTCCT-3') and Pr4 (5'-CATCCATTACATCCCGTAC-3'), which amplified a 499-bp fragment covering the HPV-16 E7 gene plus fragments of the E6 and E1 genes. Amplification of HPV-18 was performed using the primers Pr1 (5'-CCGAGCACGACAGGAACGACT-3') and Pr2 (5'-TCGTTTTCTTCCTCTGAGTCGCTT-3'), which amplified a 172-bp fragment including parts of the HPV-18 E6 and E7 genes, as described previously [42]. To ensure DNA integrity, the β-globin gene was amplified using the primers PC03 and PC04 as described previously [43]. As a positive control, PCR of DNA from SiHa (a cervical cancer-derived cell line containing one to two HPV-16 copies per cell) or HeLa (a cervical cancer-derived cell line positive for HPV-18) cells was performed concurrently with each reaction. Additionally, negative controls to assess the presence of contaminants were carried out using purified water (Gibco-BRL) and PBS instead of template DNA. Patients whose DNA sample could not be amplified were excluded from the study.

### VLPs and antigenic peptides

We previously reported the expression in tomato plants of VLPs containing the HPV-16 L1 sequence (VLP-L1) and a chimeric particle (cVLP) containing the HPV-16 L1 sequence and a string of sequences encoding T cell epitopes fused to the C-terminus, which codes for the amino acids 16-30 from E6 and 37-54, 49-57, and 86-93 from E7 [21]. VLPs used in this work were either processed from these transgenic plants or from the commercial HPV vaccine Gardasil^® ^(gVLP). VLPs derived from transgenic plants were purified by using immunoaffinity columns, as described previously [21]. Briefly, tomatoes were lysed with extraction buffer (PBS pH 7.4 with 1 mM PMSF) on ice. The homogenates were clarified by centrifugation at 10,000 rpm for 30 min at 4°C and filtered through Whatman paper No. 1 and then through a Millipore 0.8-μm filter. The supernatant was then applied to an affinity column, which was prepared by coupling sera from women vaccinated with three doses of the commercial HPV vaccine Gardasil^® ^vaccine to 4B CNBr-Sepharose (Sigma). The columns were washed with 50 vol of washing buffer (50 mM Tris-HCl, 5 mM EDTA, 150 mM NaCl, 0.1% NP-40, pH 7.4), followed by 50 vols of the same buffer without NP-40. VLPs were eluted with 100 mM HCl-glycine, pH 4.0. The VLP-containing fractions were neutralized with 100 mM Tris-HCl (pH 9.0) and dialyzed against PBS overnight. The VLPs obtained from transgenic plants was verified by transmission electron microscope, as previously reported [21].

The quantity of plant-purified VLP-L1/cVLP was measured by ELISA. For this purpose, ELISA plates (Corning) were coated with 100 μl of chromatography-purified VLP-L1 and cVLPs. Gardasil^® ^vaccine dilutions containing from 10 to 160 ng of HPV-16 VLPs/100 μL were used as standard. All samples were tested in triplicate. Plates were incubated for 16 h at 4°C, washed twice in TBS-0.1% Tween-20, blocked with 2% w/v bovine serum albumin (BSA) in TBS-0.1% Tween-20, and incubated for 2 h at 37°C. Plates were washed four times, and incubated with sera from women vaccinated with three doses of commercial vaccine at 1:500 in blocking buffer. After 2 h at 37°C, plates were washed 6 times and incubated with 1:5000 rabbit-anti-human IgG (Zymed, USA). Plates were incubated for an additional 2 h at 37°C. After eight washes, alkaline phosphatase substrate (Sigma) was diluted in a 10% diethanolamine solution (pH 9.8) and added to the plates. The absorbance at 405 nm was measured by ELISA plate reader. The concentration of VLPs was calculated by average of three repeats.

The following antigenic peptides were synthesized by Invitrogen: From HPV-16 E6 protein, 16-30 PRKLPQLCTELQTTI [44,45]; from HPV-16 E7 protein, 37-54 EIDGPAGQAEPDRAHYNI [46], 49-57 RAHYNIVTF [47], and 86-93 TLGIVCPI [44,45]. Peptides were dissolved in PBS and stored at -70°C before use. The peptide 338-346 MQYEIINYM derived from T6/GS polymerase accessory protein was used as irrelevant control [48].

### ELISA for detecting anti-VLP and anti-peptide antibodies

The specific titers of serum antibodies specific for VLPs and peptides from in subjects with LSIL and negative controls were determined by ELISA. ELISAs were performed with gVLP or VLP-L1 and cVLP obtained from transgenic plants, as well as the antigenic peptide encoding sequences contained in the C-terminus of the cVLPs, were used as antigens in ELISA assays. For this purpose, each type of VLP was diluted in PBS at 1 μg/ml, and the peptides were diluted at 5 μg/ml in sodium carbonate/bicarbonate buffer (0.1 M sodium carbonate, 0.1 M sodium bicarbonate, pH 9.6). ELISA plates were coated with 100 μl of VLPs and incubated for 16 h at 4°C. Plates were then washed four times with TBS containing 0.1% Tween 20 (TBS/Tween 20). Non-specific binding sites were blocked with 200 μl 2% BSA in TBS/Tween-20 for 2 h at 37°C. After washing, 100-μl serum samples (diluted from 1:100) were added in triplicate, and the plates were incubated at 37°C for an additional 2 h. After washing, alkaline phosphatase-conjugated rabbit-anti human IgG (Dako) was diluted in blocking buffer at 1:500 and 100 μl was added to each well. The plates were then incubated for 2 h at 37°C. After washing, alkaline phosphatase substrate Sigma 104 was diluted in a 10% diethanolamine solution (pH 9.8) and added to the plates. The absorbance at 405 nm was measured by an ELISA plate reader. The assay was considered valid only when the coefficient of variation of the triplicates was ≤10%. Additionally, all samples were tested in two wells not coated with VLPs or peptides to define non-specific reactivity. The final ELISA value was calculated by subtracting the non-specific reactivity mean absorbance from the triplicate mean absorbance.

To control for inter-assay variation, positive IgG controls selected from a previous study [22] were included in each plate and tested as described. Plates with an inter-assay coefficient of variation >10% were not considered valid. The cut-off value for high seroreactivity was determined on the basis of the absorbance distribution of the 30 positive control group samples to VLPs and peptides and was defined as the mean absorbance +3 SD after exclusion of outliers.

### Hemagglutination inhibition assay

Hemagglutination inhibition assays were performed according to Roden et al. [26]. Mouse blood was collected in a heparinized tube and erythrocytes were separated by centrifugation at 1,000 rpm for 5 min at 4°C, washed twice with PBS and suspended in dilution buffer (1% BSA in PBS) at a concentration of 1% (v/v). VLP-L1 s, cVLPs, or gVLPs (100 ng) were then incubated with various dilutions of serum samples (from 1:100 to 1:1600) at room temperature for 2 h, after which the samples were diluted with an equal volume of the 1% RBC suspension. Aliquots of the mixtures (100 μl) were transferred to a round-bottomed, 96-well plate and incubated for 3 h at 4°C. Spots indicating VLP neutralizing activity were photographed and analyzed.

### Statistical analysis

All numerical data are expressed as an average of values obtained ± standard deviation (SD), and all samples were tested in triplicate. The Wilcoxon signed-rank test and Student's t-test were used to compare the mean signal strength (absorbance) of the various groups. All tests were two-tailed and P < .05 was considered statistically significant.

## Competing interests

The authors declare that they have no competing interests.

## Authors' contributions

AMG conceived of the study and wrote the manuscript. MAGL participated in the design and production of VLPs in plants. BWS participated in discussion of results and revision of the manuscript. GPR carried out the molecular studies and cultures of tomato plants. JHM and MTG performed the ELISA assays with VLPs and peptides. KPS collected sera samples of the patients and normal donors. YTG carried out the purification of VLPs. ESO and HS obtained tissue samples and performed PCR analysis for HPV-16 infection. MLMG participated in the design and coordination of the study as well as in discussion of results. All authors read and approved the final manuscript.
